# Impact of repeated morning bright white light exposures on attention in a simulated office environment

**DOI:** 10.1038/s41598-023-35689-1

**Published:** 2023-05-30

**Authors:** Markus Canazei, Maximilian Dick, Wilfried Pohl, Johannes Weninger, Niclas Hubel, Siegmund Staggl, Elisabeth M. Weiss

**Affiliations:** 1 Department of Psychology, University of Innsbruck, Innrain 52 F, 6020 Innsbruck, Austria; 2 Research and Development Department, Bartenbach GmbH, Rinnerstrasse 14, Aldrans, Austria

**Keywords:** Environmental impact, Human behaviour

## Abstract

Attention is essential to the work. This study investigated the effects of two different light pulses on a simple attention task. In addition, the effects of subsequent exposure to constant but different illuminance levels on the continuation of the simple attention task and a subsequent complex attention task were examined. A total of 56 subjects were assigned in random order to two white light interventions that were repeated five times during the morning. Each light intervention consisted of a brief light pulse followed by constant light exposure and differed in temporal dimming dynamics and corneal illuminance. Subjective and psychometric parameters were recorded several times during light exposure. Heart rate variability (HRV) was derived from continuous electrocardiograms. Subjects showed improved reaction speed in the simple attention task, accompanied by higher HRV under a brighter light pulse without habituation by repetition. This difference in simple attention performance disappeared when light exposure remained the same after the light pulse. In addition, higher reaction speed and HRV were observed in the complex attention task under constant bright light exposure. Intermittent bright light seems promising to acutely improve attentional performance in office workplaces. Future research is needed to investigate daytime light effects on other work-related cognitive functions.

## Introduction

Alertness is a prerequisite for attention, represents a general sensitivity to incoming stimuli (external or internal)^1^, and is particularly influenced by the homeostatic sleep drive and the circadian timing system^2^. Attention, on the other hand, is a state of readiness to process specific (relevant) stimuli and typically includes an orientation phase followed by a detection and/or selection phase^3^. Alertness and especially attention can significantly affect work performance, productivity, and the risk of occupational accidents^4^.

Light exposure of rods and cones is necessary for visual perception (CIE 086: 1990). However, the human retina contains another photoreceptor type (i.e., intrinsic photosensitive ganglion cells, ipRGCs), that projects photic information directly to brain areas not associated with vision^5^. The combined projection of both photoreceptor systems is thought to be responsible for the non-visual light effects on mood, cognition, and the circadian system (reviewed in^6–8^).

There is strong evidence for non-visual light effects during the night (see, e.g.,^9,10^), but also during the evening and morning hours (i.e., 2–3 h before falling asleep and after waking, see, e.g.,^11–13^). In contrast, reports on light effects during the day are inconclusive, see, e.g.,^14^. To date, five systematic reviews have summarized daytime light effects^15–19^, suggesting a subjective alerting effect of light exposure during the day. In addition, a recently published meta-analysis^19^ provided preliminary evidence for a small but significant bright white light effect on measures of attention.

Typically, non-visual light effects are generated by continuous exposure to specific light settings. Another promising approach is exposure to intermittent light^20^. There is first evidence that nighttime intermittent light exposure exerts an acute alerting effect in the evening (e.g.,^21–23^). A complex nonlinear mechanism between all retinal photoreceptors is thought to be at play to generate these effects, with a rapid response from rods and cones and a transient response from ipRGCs, see, e.g.,^8,24–26^. Interestingly, research has also shown that ipRGCs show prolonged firing even after light offset, thus enhancing the effects of intermittent light exposure over the duration of light exposure^27^.

The present study primarily aimed at investigating the acute effects of repeated exposure to two bright white light pulses with different light doses on attentional parameters on two separate days in a simulated office environment. To avoid a reduction in visual comfort and performance, as highlighted in studies of dynamic workplace lighting^28,29^, study participants could perceive only a brief increase in illuminance during each light pulse, lasting a maximum of 30 s. Because morning bright light exposure has a higher potency to elicit non-visual light effects^15^, the study began at 7:30 AM and lasted 5 h.

In line with the review by Xu et al.^17^, we hypothesized that repeated morning exposures to bright white light pulses would increase reaction speed in a simple attention task. In addition, because the simple attention task had to be continued 5 min after the light pulse ended, we expected a beneficial carry-over effect from subsequent exposure to high corneal illuminance (i.e., the improved simple attention performance was maintained).

A second measure was complex attentional performance. The task had to be performed after the simple attention task at two constant, strongly different corneal illuminances (1360 lx and 130 lx at eye level, respectively). Again, we hypothesized that reaction speed in the complex attention task would increase at the higher corneal illuminance.

Polyvagal theory emphasizes the importance of autonomic parasympathetic activity for cognitive functions and especially for attentional processes^30^. Parasympathetic activity, as measured by HRV parameters, also reflects the ability of humans to respond adaptively to changing environmental demands^31^. Research has also shown that HRV positively correlates with attentional performance^32^. Therefore, we expected that better performance on simple and complex attentional tasks under the brighter light pulse and higher constant corneal illumination would be associated with higher HRV.

We also hypothesized that subjective sleepiness would decrease after exposure to a bright light pulse.

## Methods

### Participants

Study participants were recruited by convenience sampling (via the electronic job platform of the University of Innsbruck) and snowball sampling and had to fulfill the following criteria for study inclusion: (1) age between 18 and 65 years, (2) no self-reported sleep problems, (3) habitual sleep duration of 7–9 h, (4) no extreme chronotype and social jet lag of less than 2 h (characterized by mid-sleep points on weekdays and days off^33^), (5) absence of visual, auditory, and mental illness assessed by self-report, (6) no current medication use (other than contraceptives), (7) moderate caffeine consumption and nicotine intake and the ability to abstain from it for at least 8 h during the day, (8) willingness to wear sunglasses when outside the laboratory one day before the start of the study and after the first study day, and (9) willingness to sleep between 11:00 PM and 6:00 AM (+/− 30 min) the night before the study begins and after the first study day.

Before starting the study, a power analysis was performed using G*Power (version 3.1.9.6, University of Kiel, Germany). Assuming small to medium effect sizes of the light intervention (f = 0.15; see^19^), the inclusion of 56 subjects is necessary to detect significant differences between two light interventions with a significance level of 0.05, a power of 80%, a correlation between repeated measures of 0.50, and non-sphericity correction of 1 for a two-factor repeated-measures analysis of variance (r-ANOVA).

Data collection took place between March 15 and May 29, 2021, and subjects were financially compensated for participation in the study (total 130 €). The study protocol was approved by the Ethics Committee of the University of Innsbruck and strictly adhered to the principles of the 1968 Declaration of Helsinki. All subjects gave written informed consent before enrollment in the study.

A total of 56 subjects (75% students) were included in the study. The sociodemographic data of the sample and the ratings of the seven subjective sleep quality dimensions for both study interventions are presented in Table 1 (the sleep questionnaire is described in detail in section "Subjective ratings"). Two dependent sample t-tests failed to reveal differences in sleep quality parameters between the two study conditions (all p > 0.10).

**Table 1:** Characterization of the study sample.

Sample characteristics		
Sample size	56	
Gender distribution (female/male)	29/27	
Mean age (SD; range)	28.41 (± 7.54; 20–50 years)	
In need of visual aids (yes)	21 (38%)	
Midsleep time on workdays (SD)	03:23 AM (± 0.83 h)	
Midsleep time on free days (SD)	04:12 AM (± 1.92 h)	
Sleep duration (in hours) on workdays (SD)	7.85 (± 0.77)	
Sleep duration (in hours) on free days (SD)	8.39 (± 0.82)	

Subjective sleep quality during the study	Night before the first light pulse condition	Night before the second light pulse condition
Difficulties to fall asleep (1–5)	2.95 (± 0.91)	2.93 (± 0.98)
Difficulties to maintain sleep (1–5)	1.83 (± 0.83)	1.65 (± 0.73)
Early sleep termination (yes)	0	0
Feeling refreshed after sleep (1–5)	2.71 (± 0.79)	2.83 (± 0.77)
Mental balance before sleep (1–5)	3.40 (± 0.63)	3.53 (± 0.65)
Physical balance before sleep (1–5)	3.09 (± 0.85)	3.01 (± 0.80)
Somatic complaints during sleep (1–5)	1.25 (± 0.26)	1.26 (± 0.27)
Total sleep quality (1–5)	3.87 (± 0.57)	3.97 (± 0.62)
Total sleep duration (h)	6.94 (± 0.61)	6.82 (± 0.56)

Note: Numbers given in parentheses for subjective sleep quality indexes show either the range of the score, a binary answer category (yes), or sleep duration in hours (h).

### Study environment

**Figure 1:**
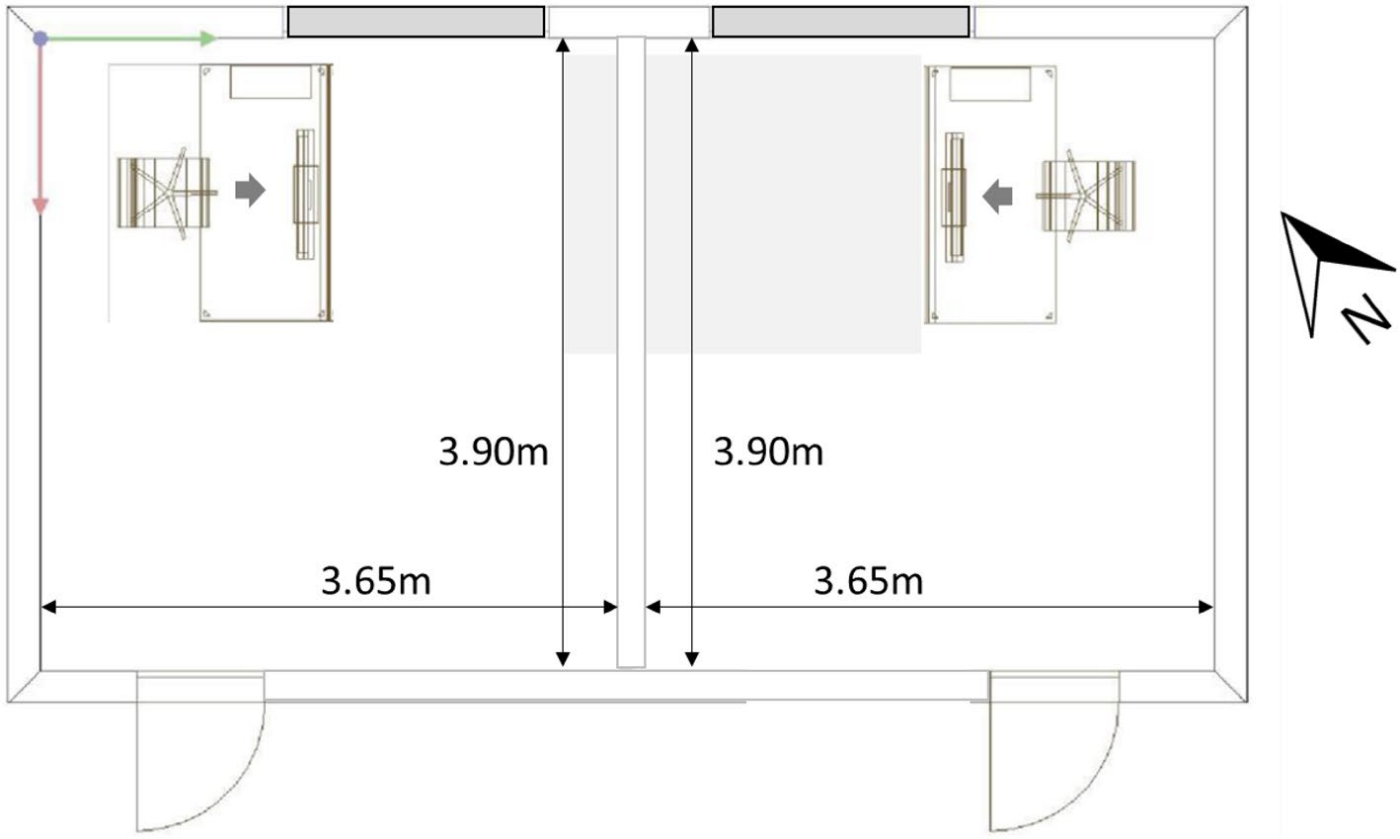
Schematic plan of the study location (grey bars indicate the windows of the test rooms).

Two identical study rooms (Fig. 1), each with white painted walls and ceilings (rho = 0.85) and a grey carpet (rho = 0.35), were each set up with a computer workstation. Study participants run through the study protocol on two consecutive days, beginning at 7:00 AM and ending after 6 h in the early afternoon (around 01:00 PM). The windows of the test rooms faced northeast and were covered with blinds with low light transmittance (< 5%), which minimized daylight penetration.

Room temperatures were not monitored during the study, but were measured once in a pilot test before the study began and ranged from 22 to 24 °C. To freshen the air during the study, windows were tilted and doors were opened during a 15-min study break starting at 09:30 AM, during which participants were allowed to consume decaffeinated beverages or leave the study room for a bathroom break. Smoking and socializing were not permitted during the break.

### Light interventions

Four ceiling luminaires (research prototypes developed by Bartenbach GmbH, Austria) provided ambient lighting for the test rooms. Each luminaire contained 48 warm-white LEDs (Luxeon Z-ES, 2219 K) and cold-white LEDs (Luxeon Z-ES, 5626 K) which together provided neutral-white light (4026 K; color rendering index: 93; CIE x/y coordinates: 0.379/0.374). Ambient lighting was maintained at a low level throughout the study (100 lx horizontal, at floor level) and met current indoor lighting standards (e.g., unified glare rating < 19, color rendering index > 80).

**Figure 2:**
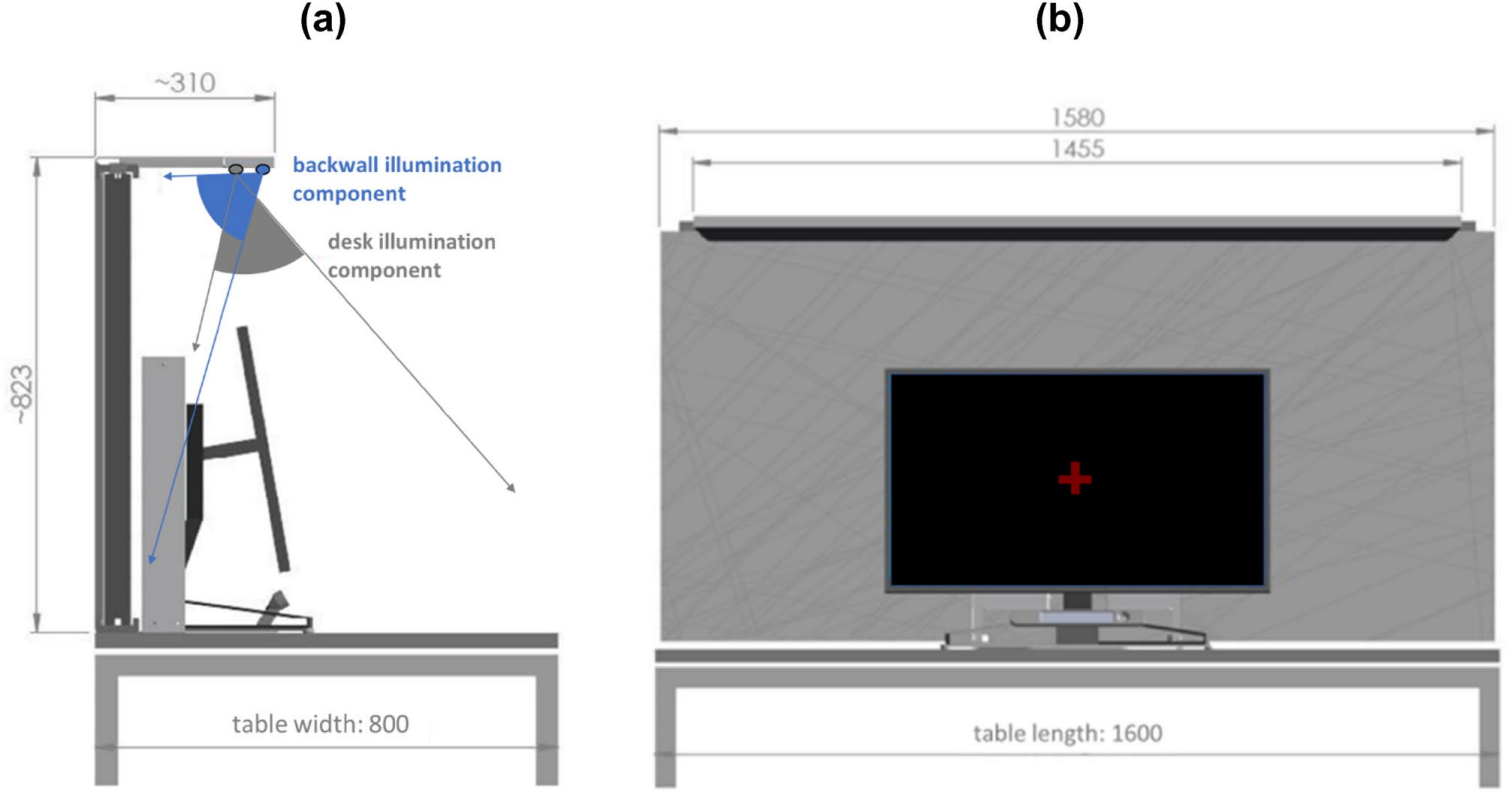
Workplace luminaire—profile view (**a**) and frontal view (**b**).

The room lighting was supplemented by a task luminaire (research prototype developed by Bartenbach GmbH, Austria, as part of the EU-funded ‘REPROLIGHT’ project; see https://cordis.europa.eu/project/id/768780/de). The luminaire consisted of 144 free-form aluminium reflectors that separately illuminated the desk and the backwall of the workstation (Fig. 2a). Each reflector contained a warm-white LED (Luxeon Z-ES, 2219 K, color rendering index: 88; CIE 1931 x/y coordinates: 0.504/0.289) and a cold-white LED (Luxeon Z-ES, 5626 K, color rendering index: 87; CIE 1931 x/y coordinates: 0.329/0.358). By using this reflector technology, the light from the LEDs was perfectly shielded (i.e., subjects could not see the light sources either directly or through an opaque surface from any working position and viewing direction) and luminance levels at the workstation were kept below 1500 cd/m^2^ (measured with a spot luminance meter; Konica Minolta LS-150). Both measures were intended to avoid glare and distraction^34^. The luminaire achieved an overall output of 101 lumens per watt and was mounted on a small pole, at a height of 154 cm above the floor level. In addition, a reflective white textile backwall (rho > 80%) was integrated into the pole (see the gray background in Fig. 2b) and both the pole and backwall were placed at the desk behind the monitor (see Fig. 2a).

**Figure 3:**
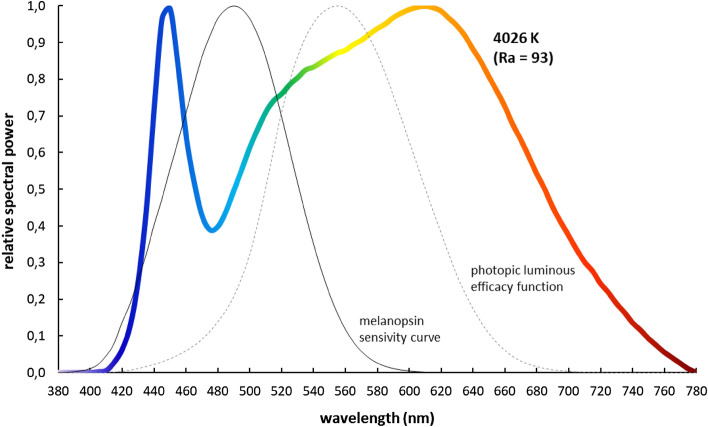
Relative spectral power distribution of the light intervention.

In the present study, the color temperature of lighting was kept constant at 4026 K (color rendering index: 93; CIE x/y coordinates: 0.379/0.374). Spectral power distribution (see Fig. 3; data are given in Supplementary Material S1) and illuminances at eye level were measured with a calibrated spectroradiometer (JETI specbos 1211-2; JETI Technische Instrumente GmbH, Germany). Following current recommendations (CIE S 026:2018; Metrology of optical radiation for ipRGC-influenced responses to light), further photometric measures of the light interventions are summarized in Table 2.

**Table 2:** Photometrical characterization of the light conditions.

	Standard lighting	Stronger light pulse condition (maximum)	Weaker light pulse condition (minimum)	Weaker light pulse condition (maximum)
Alpha-opic equivalent daylight (D65) illuminance (lx) at eye level				
S-cone	78	811	39	519
M-cone	115	1207	58	772
L-cone	130	1361	65	871
Rhodopsin	94	984	47	629
Melanopsin	86	900	43	576
Classical photometrical measures				
Illuminance at eye level (lx)	130	1360	65	870
Horizontal illuminance at desk level (lx)	520	5440	260	3480
Irradiance at eye level (W.m-2)	0.46	4.78	0.23	3.06

Note: measurements at eye level were done in a fixed position (i.e., 120 cm above floor level at the edge of the table with a horizontal viewing direction); measurements at desk level are the average of nine spot measurements equally distributed across the desk surface.

Under the stronger light pulse condition, task illumination was initially increased from a corneal illuminance of 130 lx (86 m-EDI) to 1360 lx (900 m-EDI) over a 30-s period and remained there for 29.5 min. To generate a similar perceived sudden increase in task illumination in the weaker light pulse condition, illuminance was first decreased before being rapidly increased. Thereafter, corneal illuminance was to be imperceptibly dimmed to 130 lx over a period of several minutes. By using these lighting control strategies, the two light pulse conditions should be perceived as equal. To derive the appropriate dimming dynamics for the weaker light pulse condition, a small pilot study was conducted within the project team (n = 5 subjects). Finally, the weaker light pulse condition was specified as follows: illuminance was first reduced by 50% to 65 lx at eye level within 30 s and then immediately increased to 870 lx at eye level (576 m-EDI) within 15 s (see Table 2); subsequently, illuminance was gradually reduced to standard task lighting (130 lx at eye level) over a period of 3.25 min and kept at this level. Overall, the two light pulses differed in their temporal dimming dynamics and light dose (i.e., light dose was reduced by a factor of 3.38 under the weaker light pulse compared with the stronger light pulse) and thereafter provided constant but different corneal illuminances (130 lx and 1360 lx, respectively).

As discussed earlier^35^, implementing an ineffective white light control condition is hard to achieve since dim light is easy to detect. Therefore, we decided to use two different light pulse strategies in our study instead of one active and one inactive light condition. Because the abrupt and perceivable increase in task illumination (i.e., light pulse) occurred when subjects were performing a simple attentional task in a highly concentrative status, this parallel implementation further hampered the ability to detect a difference between the two light pulse settings.

### Outcome measures

#### Psychometric tests

Attention describes the ability to select specific (environmental) information and is fundamental to work performance. In the present study, simple sustained attention performance was quantified with an auditory psychomotor vigilance task (aPVT;^36^). Short auditory cues (1000-Hz tones with a duration of 200 ms) were presented via headphones at variable intervals (2–10 s). Participants were instructed to keep their eyes open and press the space bar on a standard keyboard (operating at 60 Hz) as quickly as possible when they heard the stimulus. No visual feedback on reaction time was displayed on a black screen (< 10 cd/m^2^). The aPVT version in our study lasted longer than usual (15 min instead of 5 or 10 min^37^) and included 120 auditory stimuli. The main outcome parameter was reaction speed (inverted reaction time, 1/sec) for responses between 100 and 500 ms. A second outcome parameter was the number of lapses (responses after 500 ms). The outcome parameters were derived for three periods of 5 min each (’baseline’: first 5 min of aPVT; ’intervention’: between 5 and 10 min of aPVT; ’different light’: last 5 min of aPVT). This procedure allowed us to quantify the light effects on the simple attention task separately for the light pulses and the constant but different corneal illuminance levels.

Response inhibition is the ability to respond to a target stimulus, but suppressing a motor response to a distractor stimulus, requires a high level of attention, is part of the executive control system, and thus can be described as a complex attentional task. To measure the effects of repeated prolonged bright light exposure on complex attention, a visual go/no-go task (GNT) was performed in the present study^38^. In this test, an equal number of green and blue rectangles with different orientations (horizontal and vertical) were shown on a monitor with a black background (< 10 cd/m^2^). The green figures were the target stimuli and the blue figures were the distractor stimuli. After subjects fixated a white cross on the screen for 800 ms, a black screen was shown for 300 ms. Then, one of four rectangles (green-vertical, green-horizontal, blue vertical, blue horizontal; in equal proportions and in random order) was presented for 1000 ms. Subjects had to press the spacebar when the green rectangle (in any orientation) was perceived, or otherwise suppress the motor response. After the rectangle presentation ended, subjects again saw a black screen for 300 ms before the next cycle of stimulus presentation began. In total, 250 rectangles were shown in 10 min. The main outcome parameter was the reaction speed for correct responses, which were given between 100 and 1000 ms. Because of the right-skewed distribution of recorded reaction times, reaction speed was calculated as inverted reaction time. The secondary outcome parameter was the number of commission errors (i.e., the number of responses to blue rectangles between 100 and 1000 ms).

Visual acuity refers to the ability to discriminate small visual details and depends on both the quality of the ocular medium (e.g., refractive error of the lens) and the task illumination. Visual acuity increases with higher illuminance, but glare has a negative effect on visual perception at high illuminance levels^39^. The Freiburg Visual Acuity Test (FrACT^40^) was used to measure visual acuity at the end of each study day at low and high corneal illuminance levels (130 lx and 1360 lx, respectively). In the test, subjects had to indicate the apertures of Landolt rings of different sizes with the corresponding keyboard arrows for about 15 min. Depending on the ratio of correct responses in the previous trials, the size of the Landolt rings changed dynamically over time. Visual acuity was then derived from the visual angle of the smallest reliably distinguishable aperture of the Landolt rings and described as the Logarithmic Minimum Angle of Resolution (LogMAR).

#### Subjective ratings

Subjective ratings of alertness correlate with objective measures of alertness derived from electroencephalography^41^ and are therefore frequently used in non-visual light impact research. Several reviews have confirmed a positive correlation between bright white light exposure and increased subjective alertness, see for example^15,16^. In the present study, a 5-point, bipolar visual analog scale (VAS) was used to assess the current subjective sleepiness-alertness state immediately before and after the end of performing the simple attention task. The scale included the five response options: ‘sleepy but no effort to stay awake’ (1), ‘some signs of sleepiness’ (2), ‘neither alert nor sleepy’ (3), ‘fairly alert’ (4) and ‘alert’ (5).

Perceived workload influences task performance and results from a complex interaction between task-related demands, personal factors (e.g., skills and motivation), and contextual factors^42^. To quantify perceived workload at the end of each of the five light interventions, the first part of the NASA Task Load Index (NASA TLX^43^) was used. Subjects had to rate their workload on six dimensions (mental demand, physical demand, temporal demand, performance, effort, and frustration) on a scale of 0 to 100, divided into increments of five. The six ratings were averaged to an overall task load score.

Mood states can be acutely altered by bright light exposure or variable lighting conditions (see, e.g.,^44,45^). In the present study, mood state was assessed using the Positive and Negative Affect Schedule (PANAS^46^) at the end of each of the five light interventions at constant but varying corneal illuminance levels. The PANAS includes 20 adjectives, ten of which are associated with positive affect dimensions (e.g., attentive, active, excited) and 10 of which are associated with negative affect dimensions (e.g., hostile, nervous, afraid), respectively. Adjectives were rated on a 5-point Likert scale (1—‘not at all’ to 5—‘very much’), and added to the total scores for each affect dimension.

Asthenopic symptoms are typically caused by sustained concentrated visual effort, acute bright light exposure, or exposure to flickering light^47^. A brief ten-item questionnaire, selected from the 19-item clinical asthenopia questionnaire^48^, was used once at the end of each study day under each light intervention to measure a range of visual complaints (eye strain, headache, watery eyes, itchy eyes, burning eyes, blurred vision, pain around and in the eyes, glare, dizziness, and nausea). Items were rated on a 6-point Likert scale (1—‘not at all’ to 6—‘very’) and summed for a total score for visual complaints ranging from 10 to 60.

A sleep questionnaire^49^ with 32 items was used to measure sleep quality on seven dimensions (difficulty falling asleep; difficulty maintaining sleep; early termination of sleep; feeling refreshed after sleep; mental balance before falling asleep; physical balance before falling asleep; somatic complaints during sleep) during the last night before each study day. Based on the ratings (5-point Likert scale), an overall index of sleep quality was determined. Additionally, the questionnaire asked about falling asleep and staying asleep the previous night to calculate sleep duration. Study participants completed the sleep questionnaire immediately after awakening on the two subsequent study days.

#### Physiological reaction to light exposure

Measures of heart rate variability (HRV) reflect the activity of the sympathetic and parasympathetic branches of the autonomic nervous system^50^ and have been used to assess workload, physiological stress response, and stress recovery^51^. Interestingly, HRV reflects vigilant attention in sleep-restricted subjects^52^ and is associated with increased performance on tasks requiring sustained attention^53^.

To study the light-induced cardiovascular responses, an electrocardiogram was continuously recorded on each study day using the portable device ‘Mega Faros 180’ (Bittium Corporation, Finland). The ‘Mega Faros 180’ is waterproof (IP 67), lightweight (13 g), tiny (48 × 29 × 12 mm), and provides a high-resolution (up to 1024 Hz) single-channel electrocardiogram (ECG). In the present study, the electrocardiogram was stored at a sampling frequency of 1000 Hz. During preparations at the beginning of this study, we noted an inaccuracy of the real-time clock of the ECG device of several seconds when data were recorded over a period of six or more hours. As countermeasures, we (1) synchronized the ECG device clock with the computer clock by initializing it at the beginning of each study day and (2) instructed study participants to press a marker button on the device at the beginning of each cognitive test to quantify the discrepancy in the clock times of the ECG and attentional test data streams. Raw data were imported into the software ‘Cardioscope Analytics’ (Smart Medical, Gloucestershire, United Kingdom) and analyzed using a 5-min moving-window technique, with heart rate data analyzed in 5-min windows that followed each other in 30-s epochs. For each epoch, a quality index was generated by the software (incorporating beat and arrhythmia abnormalities). In the present study, a quality index of at least 95% was set as the lower threshold. To determine HRV, the R-waves of the QRS signal in the electrocardiogram were first detected by the software, and then the time between two consecutive R-waves (RR interval; in milliseconds) was determined. Based on the RR intervals, the root mean squared of successive difference in RR intervals (RMSSD) was determined as a robust indicator of HRV.

Based on the gliding 5-min window technique, RMSSD data for continuous 30-s epochs were available and had to be prepared for data analysis. To examine the temporal dynamics of physiological responses during the performance of aPVT, HRV parameters were first averaged for each subject over the three separate 5-min periods of aPVT (termed ‘baseline’, ‘intervention’, and ‘different light’) and these data were subjected to statistical analyses. For the 10-min GNT, the individual HRV parameters derived from the central 5-min test periods (minute 2.5 to minute 7.5) were averaged for each subject and then subjected to statistical analyses. A similar procedure was applied to the HRV parameters derived from the five 25-min resting periods. Here, the individual RMSSD data from the central 20-min period (minute 2.5 to minute 22.5) were averaged and then subjected to statistical analyses.

### Study protocol and lighting control strategy

Before the study started, a randomization list was generated (www.randomizer.org) to assign study participants to the two interventions.

Subjects entered the test room illuminated with standard lighting (see Table 2) at 07:00 AM. In the following 30 min, subjects handed in their sleep quality questionnaire and performed both cognitive tests for training purposes on each study day. Under the instruction of the study coordinator, subjects then mounted three adhesive ECG electrodes to their chest and started ECG recordings. At 07:30 AM, the five test cycles (Fig. 5), each lasting on hour, began (test cycle 1: 07:30 AM–08:30 AM; test cycle 2: 08:30 AM–09:30 AM.; test cycle 3: 09:30 AM–10:30 AM; test cycle 4: 10:30 AM–11:30 AM; test cycle 5: 11:30 AM–12:30 PM).

In each test cycle, the psychometric tests (performance tests and questionnaires) were performed on the computer, and the light interventions were automatically started by a computer program. In this way, the contact time between the study administrator and the study participants was minimized and the start of the light intervention and the psychometric measurements were synchronized.

In each test cycle, subjects were initially exposed to standard lighting for 25 min. During this time, they were allowed to read available newspapers and magazines, were instructed to keep their eyes open, and could see a clock on the screen counting the time to the start of the upcoming psychometric test. After 25 min, subjects first rated their state of alertness (VAS), and then the aPVT began. After 5 min, while the aPVT was performed in a highly concentrated manner, the task lighting automatically switched to one light intervention setting. Study participants continued with the aPVT for an additional 10 min.

Ten minutes after the start of the two light interventions, the aPVT ended and subjects reassessed their alertness state (VAS). Then, the complex attention test (GNT) started automatically. In the first light pulse condition, this test was performed under bright light exposure (1360 lx at eye level) whereas in the second light pulse condition, the test was performed under standard task lighting (130 lx at eye level).

After completion of the GNT, study participants rated their affective state (PANAS) and workload (NASA TLX) under the two different corneal illuminance levels. Then, the illuminance under the stronger light pulse condition was dimmed to standard illumination (130 lx at eye level) for a period of 1 min, and the 1-h test cycle ended.

**Figure 4:**
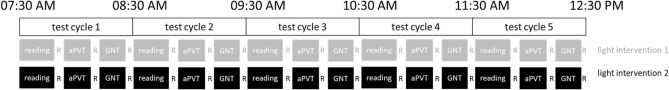
Timeline of the entire experiment. R, different rating scales; aPVT, auditory psychomotor vigilance task; GNT, go/no-go task.

Subjects run through five test cycles on each of the two subsequent study days (Fig. 4). At the end of the last test cycle (12:30 PM), the illuminance levels at the workstation were not changed, and study participants rated their visual complaints and performed the computerized visual acuity test (FrACT) under both study conditions (130 lx and 1360 lx, respectively). Subjects left the study room at approximately 01:00 PM.

To minimize the influence of daylight light exposure, the study began in the early morning (07:00 AM), and subjects were instructed to wear sunglasses outdoors the day before the study began and in the afternoon between the first and second study day.

**Figure 5:**
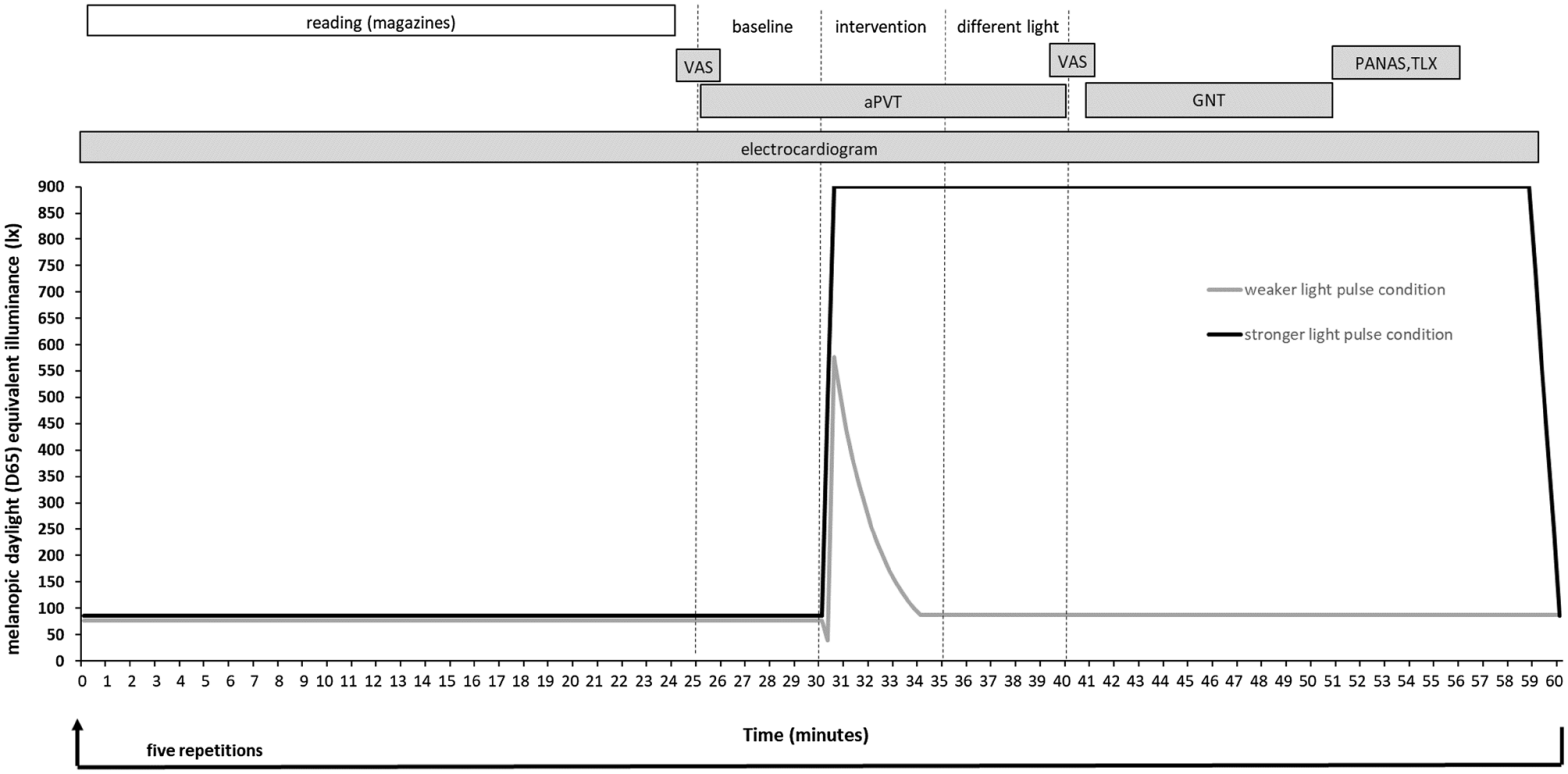
Study protocol and lighting control strategies within the 1-h test cycle. aPVT, auditory psychomotor vigilance task; GNT, go/no-go task; VAS, visual analog scale; PANAS, positive and negative affect scale; TLX, NASA task load.

The study protocol and light control strategies within a test cycle are shown in Fig. 5.

### Data analysis

For descriptive analyses, mean (M) and standard deviation (SD) were calculated. All graphs and descriptive ANOVA results show means and 95% confidence intervals (CI) of means unless otherwise noted.

For statistical testing of the hypotheses, two-factor r-ANOVAs were performed with the factor ‘light intervention’ (2 levels: stronger light pulse, weaker light pulse) and the time-of-day factor ‘test cycle’ (5 levels: test cycle 1–5) for each of the three parts (‘baseline’, ‘intervention’, and ‘different light’) of the aPVT reaction speed and GNT reaction speed, the PANAS positive and negative affect scale, and the NASA TLX. For VAS, a three-factor r-ANOVA was performed (factors: ‘pre-/post aPVT measurement’; ‘light intervention’ and ‘test cycle’). Before performing all analyses of variance, data were checked for outliers, normality, and sphericity, and in case of violation of sphericity, a Greenhouse–Geisser correction of the significance level was applied. All pairwise post hoc comparisons were performed with Bonferroni-corrected significance levels. To avoid a possible error caused by the application of three r-ANOVAs separately for each 5-min period of the aPVT, a full three-factorial r-ANOVA model with the factors ‘light intervention’, ‘test cycle’ and ‘parts’ was also run for aPVT reaction speed. This analysis confirmed the results of the three separate two-factor r-ANOVAs and is reported in Supplementary Materials S2.

Data of lapses in the aPVT and commission errors in the GNT violated the assumption for applying r-ANOVAs. Therefore, non-parametric Friedman ANOVAs were first run separately for each intervention to identify potential effects of the test cycle. If the Friedman ANOVA indicated a significant effect, pairwise Wilcoxon tests with Bonferroni-corrected significance levels were then calculated. Furthermore, for each test cycle, differences between the two interventions were calculated by applying Wilcoxon tests with Bonferroni-corrected significance levels for multiple comparisons (alpha = 0.01).

Individual HRV data had to fulfill a quality criterium (delivered by the software ‘Cardioscope Analytics’) to be included in the statistical analyses. If the quality fell below 95%, the HRV data were replaced with the last observation of the subject carried forward. Data replacement was required for 5.0% of all recordings for the aPVT and for 6.4% for the GNT. HRV data were analyzed for the three parts (‘baseline’, ‘intervention’, and ‘different light’) of the aPVT and the entire 10-min period of the GNT. To control for possible carry-over effects of the light interventions, HRV data for the five 25-min resting periods were also analyzed.

The effects of the intervention on perceived visual complaints, FrACT, and subjective sleep quality were determined by t-tests for two dependent samples.

A confounder analysis was performed on physiological states during resting periods. We also compared reaction speeds in the two attention tasks between the first and second study day. The results are presented in Supplementary Materials S3.1 and S3.2. Furthermore, we investigated gender differences in performance on the simple and complex attention tests, as well as heart rate variability data while performing these tests. Statistical analyzes revealed no significant influence of the factor of gender (results are shown in Supplementary Materials S4).

All data analyses were performed using the software SPSS (IBM SPSS Statistics for Windows, version 28.0) with a 5% significance level and two-sided testing. Effect sizes in r-ANOVAs are shown as partial eta-squared values (η^2^_p_) indicating small (0.01 ≤ η^2^_p_ < 0.06), moderate (0.06 ≤ η^2^_p_ < 0.14), or large effect sizes (η^2^_p_ ≥ 0.14).

## Results

### Simple attention performance (aPVT), heart rate variability, and changes in subjective sleepiness-alertness ratings

#### Period with no difference in light levels (‘baseline’)

A two-factorial r-ANOVA revealed neither a significant interaction between the factors ‘light intervention’ and ‘test cycle’ nor main effects of either factor on the aPVT reaction speed (interaction: *p* = 0.978; light condition: *p* = 0.218; test cycle: *p* = 0.407).

The number of lapses in aPVT also did not differ between the two light interventions across the five test cycles (stronger light pulse condition: *p* = 0.583; weaker light pulse condition: *p* = 0.501). Pairwise comparisons between the two study interventions also revealed no difference in the number of lapses for each test cycle (all *p* > 0.10).

Moreover, we observed a significant main daytime effect for RMSSD, *F*(4, 220) = 8.425, *p* < 0.001, η^2^_p_ = 0.133. Pairwise comparisons revealed higher RMSSD in test cycle 2 (58.6 ± 5.7) compared to test cycle 1 (52.3 ± 5.4; *p* < 0.001), test cycle 4 (49.9 ± 5.3; *p* < 0.001) and test cycle 5 (51.3 ± 5.1; *p* = 0.002). No other effects of the r-ANOVA were significant on RMSSD while performing the aPVT during the ‘baseline’ period (interaction: *p* = 0.945; light intervention: *p* = 0.341).

#### Period during light pulses (‘intervention’)

**Figure 6:**
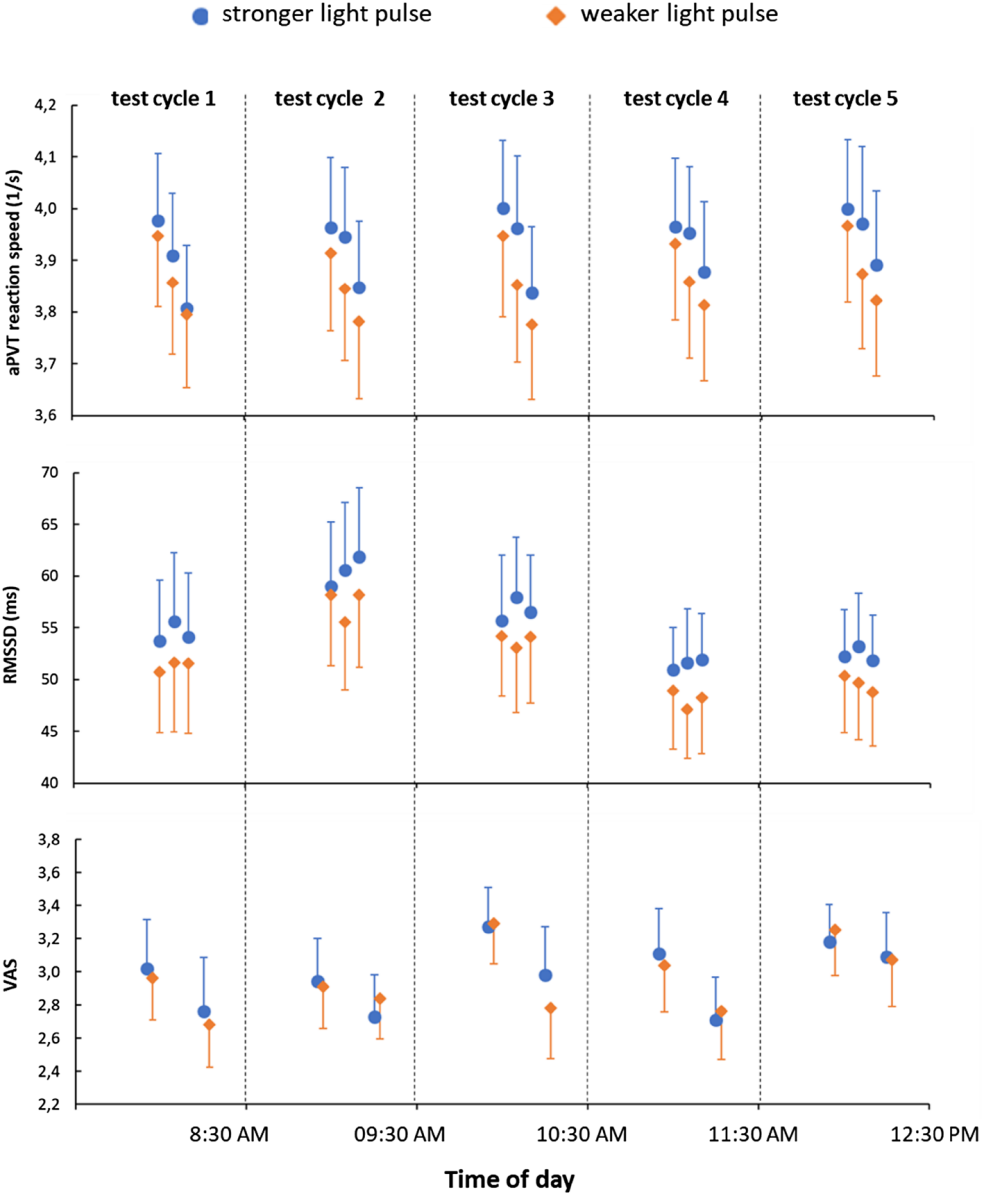
aPVT reaction speed, RMSSD, and pre-/post aPVT sleepiness-alertness ratings (VAS). Note: the figure shows means and 95% confidence intervals of the means.

We did not observe a significant interaction or main effect of test cycle in the reaction speed of aPVT, *p* = 0.815 and *p* = 0.682, respectively. However, the main effect of light intervention was significant, *F*(1, 55) = 8.995, *p* = 0.004, η^2^_p_ = 0.141, with a shorter reaction speed under the stronger light pulse (3.95 ± 0.13) compared with the weaker light pulse (3.86 ± 0.13), see Fig. 6, first panel. The number of lapses in the aPVT was not different between the two light pulses across the five test cycles (first light pulse condition: *p* = 0.507; second light pulse condition: *p* = 0.508). Pairwise comparisons between the two interventions also revealed no significant difference in the number of lapses for each test cycle (all *p* > 0.10).

Similar results were found for the HRV-parameter RMSSD in the period ‘intervention’ of the aPVT. We observed a significant main effect of light intervention (*F*(1, 55) = 5.177, *p* = 0.027, η^2^_p_ = 0.086), with higher RMSSD under the stronger light pulse (55.8 ± 5.3) compared to the weaker light pulse (51.4 ± 5.5), and a significant daytime effect (*F*(4, 220) = 9.834, *p* < 0.001, η^2^_p_ = 0.152). Post-hoc tests revealed significantly higher RMSSD values in test cycle 2 (58.1 ± 5.8) compared to test cycle 1 (53.6 ± 6.2; *p* = 0.010), test cycle 4 (49.4 ± 5.6; *p* < 0.001), and test cycle 5 (51.4 ± 4.9; *p* < 0.001). Moreover, RMSSD was higher in test cycle 3 (55.5 ± 5.2) than in test cycle 4 (*p* < 0.001) and test cycle 5 (*p* = 0.014), see Fig. 6, middle panel. The interaction effect on RMSSD between the two factors did not reach significance (*p* = 0.949).

#### Period with constant but different light levels (‘different light’)

We did not observe a significant interaction nor main effects of the two factors in reaction speed of the aPVT during the ‘different light’ period (interaction: *p* = 0.606; factor ‘light intervention’: *p* = 0.057; factor ‘test cycle’: *p* = 0.115). However, the number of lapses during the study varied in the last 5-min period of the aPVT under the higher corneal illuminance (χ^2^(4) = 9.665, *p* = 0.046). Post-hoc analysis revealed a higher median number of lapses in the fourth cycle (*Mdn* = 3.0) compared to the first, second, third, and fifth cycles (all *Mdn* = 2.0; all *p* < 0.05). Under the lower corneal illuminance, the number of lapses was also different across the study day (χ^2^(4) = 9.599, *p* = 0.048). Again, lapses were higher in the fourth cycle (*Mdn* = 3.0) than in the other four test cycles (all *Mdn* = 2.0; all *p* < 0.05). Finally, a pairwise comparison between the light interventions in each test cycle revealed no differences in the number of lapses in the period with constant but widely varying corneal illuminances (all *p* > 0.10).

Statistical analysis further revealed a significant time-of-day effect in RMSSD, *F*(4, 220) = 13.360, *p* < 0.001, η^2^_p_ = 0.195. This HRV parameter was also significantly higher in test cycle 2 (60.0 ± 6.1) than in test cycle 1 (52.8 ± 5.7; *p* < 0.001), test cycle 4 (50.1 ± 4.6; *p* < 0.001), and test cycle 5 (50.3 ± 4.5; *p* < 0.001). Moreover, the two-factor r-ANOVA revealed neither a significant interaction nor a main effect of light intervention on RMSSD in the period with different corneal illuminance levels (interaction: *p* = 0.949; light intervention: *p* = 0.147).

#### Changes in subjective sleepiness-alertness ratings

For the rating scale, a three-factorial r-ANOVA was performed with the factors ‘light intervention’, ‘test cycle’, and ‘pre-/post aPVT measurement’. The statistical test revealed a significant interaction effect between the factors ‘pre-post aPVT measurement’ and ‘test cycle’, *F*(4, 216) = 3.058, *p* = 0.023, η^2^_p_ = 0.054, see Fig. 6, last panel. Post-hoc tests yielded increased subjective sleepiness scores independent of the light intervention after performing the aPVT in cycle 1, cycle 3 and cycle 4 (*p* < 0.001, *p* < 0.001, and *p* = 0.002, respectively). However, neither the three-factor interaction (*p* = 0.092), nor the two-factor interactions between the factors ‘light’ and ‘pre-post aPVT measurement’ (*p* = 0.295) and the factors ‘light intervention’ and ‘test cycle’ (*p* = 0.439) reached significance.

### Complex attention performance (GNT) and heart rate variability under different but constant light levels

#### GNT reaction speed

**Figure 7:**
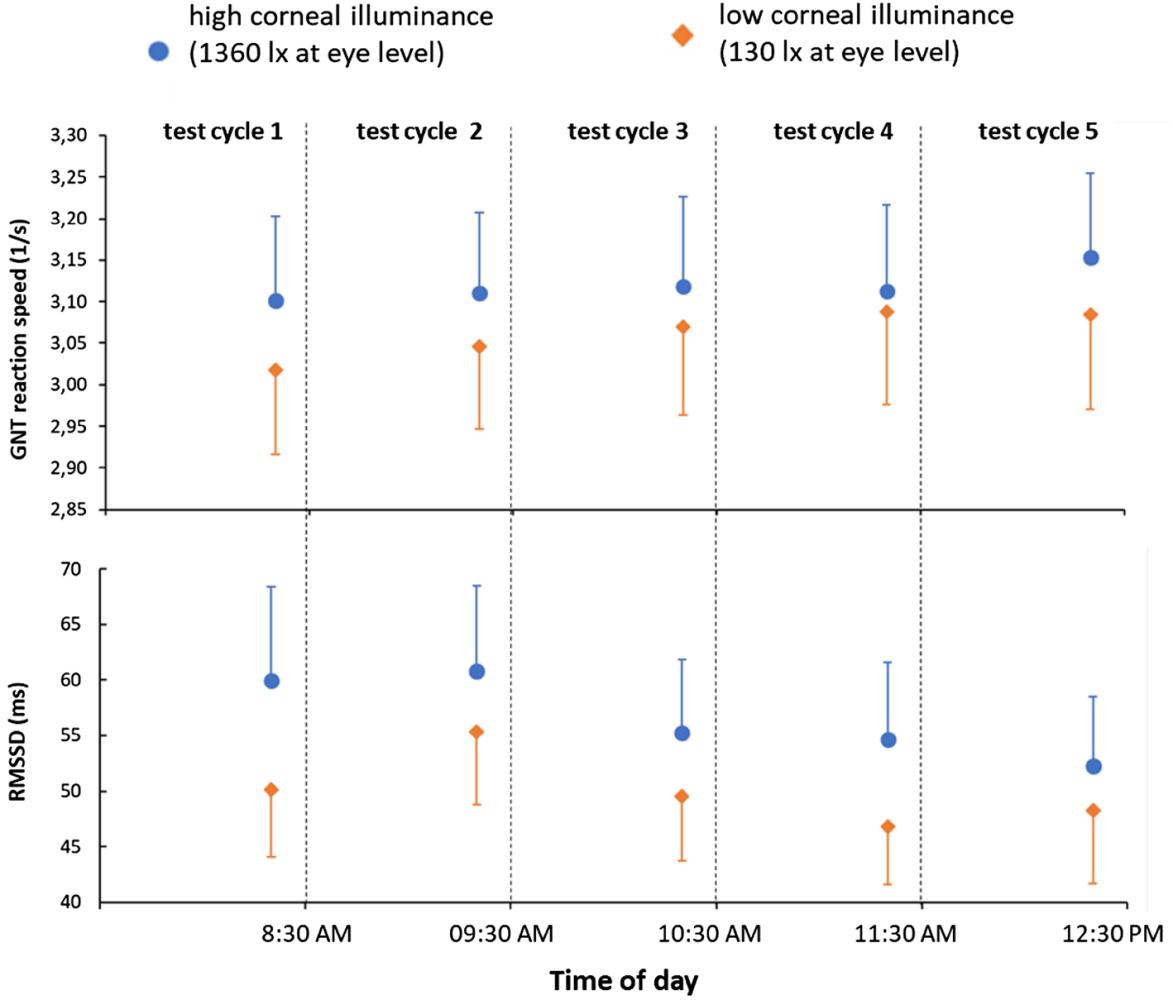
GNT reaction speed and cardiovascular parameters RR-intervals and RMSSD in the GNT. Note: the figure shows means and 95% confidence intervals of the means.

A two-factor r-ANOVA found no significant interaction effect between the factors ‘light intervention’ and ‘test cycle’ in the GNT reaction speed, *p* = 0.423. However, significant main effects were found for the light intervention (*F*(1, 55) = 7.710, *p* = 0.007, η^2^_p_ = 0.123), with faster reaction speed under high corneal illuminance (3.12 ± 0.10) compared to low corneal illuminance (3.06 ± 0.10), as well as for the daytime period (*F*(4, 220) = 3.707, *p* = 0.011, η^2^_p_ = 0.063). Bonferroni-corrected post-hoc tests revealed a significant difference in GNT reaction speed between test cycle 1 (3.06 ± 0.10) and test cycle 5 (3.12 ± 0.10); *p* = 0.037, see Fig. 7, first panel.

#### GNT commission errors

Commission errors in GNT varied during the study day at high corneal illuminance (χ^2^(4) = 28.739, *p* < 0.001). Post-hoc analysis revealed higher median commission errors in test cycle 3, test cycle 4, and test cycle 5 (all *Mdn* = 3.0) compared to test cycle 1 (*Mdn* = 1.5) and test cycle 2 (*Mdn* = 2.0; all *p* < 0.05). At low corneal illuminance, commission errors were also different during the day (χ^2^(4) = 17.415, *p* = 0.002). Again, median commission errors were higher in test cycle 3, test cycle 4, and test cycle 5 (all *Mdn* = 3.0) than in test cycle 1 (*Mdn* = 2.0; all *p* < 0.05). Finally, pairwise comparisons between the two corneal illuminances for each test cycle revealed no differences in commission errors (all *p* > 0.10).

#### Heart rate variability while performing the GNT

A two-factorial r-ANOVA found significant main effects of light intervention and test cycle for RMSSD during the GNT (light intervention: *F*(1, 55) = 7.996, *p* = 0.007, η^2^_p_ = 0.127; test cycle: *F*(4, 220) = 7.356, *p* < 0.001, η^2^_p_ = 0.118). RMSSD was higher at high corneal illuminance (56.6 ± 5.5) than at low corneal illuminance (50.0 ± 5.5), see Fig. 7, second panel. Moreover, pairwise comparisons revealed specific time-of-day effects, i.e., a higher RMSSD value in test cycle 2 (58.1 ± 6.4) compared to test cycle 3 (52.4 ± 5.3; *p* = 0.019), test cycle 4 (50.8 ± 5.5; *p* = 0.001) and test cycle 5 (50.2 ± 6.1; *p* < 0.001). No significant interaction effect was found for RMSSD during the GNT (*p* = 0.364).

### Further results

#### Affective state at the end of each test cycle

**Figure 8:**
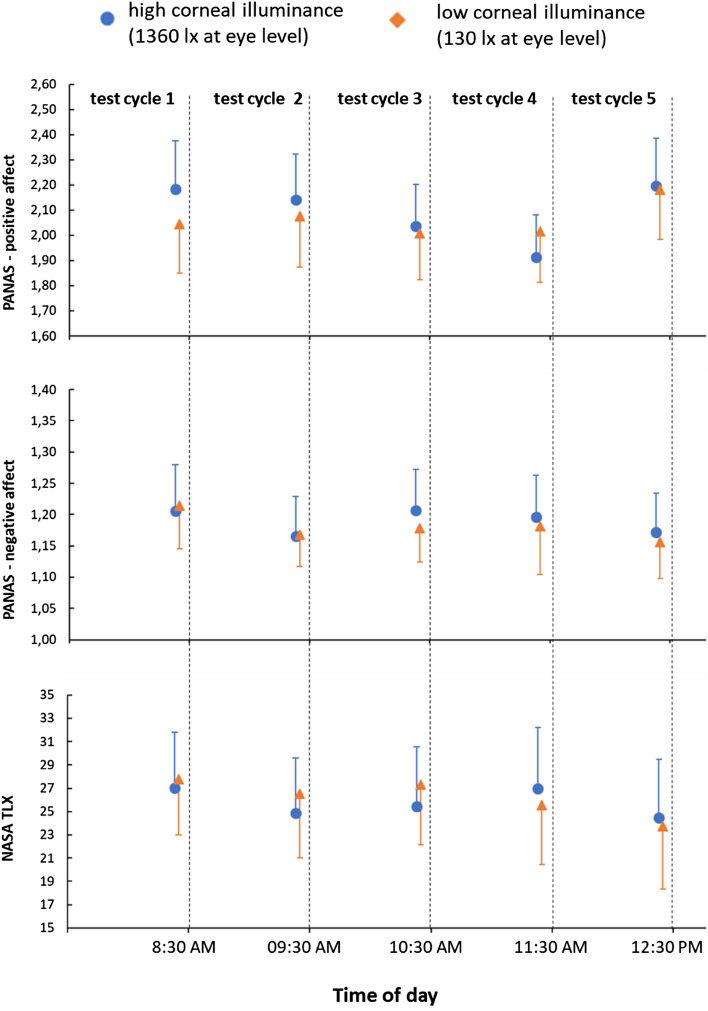
Positive and negative affect state and perceived workload (NASA TLX). Note: means and 95% confidence intervals of the means are shown.

Two-factor r-ANOVAs were conducted separately for the positive and negative affect dimensions of the PANAS, which were scored five times at the end of each test cycle. Neither the interaction nor the main effect of the light intervention reached significance for the positive affect state (interaction: *p* = 0.126; light intervention: *p* = 0.455). However, we observed significant time-of-day effects, *F*(4, 220) = 7.131, *p* < 0.001, η^2^_p_ = 0.115. Post-hoc comparisons revealed higher positive affect in test cycle 5 (2.2 ± 0.2) compared to test cycle 3 (2.0 ± 0.2; *p* = 0.011) and test cycle 4 (1.9 ± 0.2; *p* < 0.001), see Fig. 8, first panel.

We did not find a significant result for the PANAS dimension of negative affect (interaction: *p* = 0.870; test cycle: *p* = 0.097; light intervention: *p* = 0.618), see Fig. 8, second panel.

#### Subjective workload at the end of each test cycle

A two-factorial r-ANOVA found a significant main daytime effect in the perceived workload of the NASA TLX, *F*(4, 220) = 2.833, *p* = 0.041, η^2^_p_ = 0.049, see Fig. 7. However, Bonferroni-corrected post-hoc tests showed no significant differences between any two test cycles. Moreover, neither the interaction effect nor the main effect of the light intervention was significant (interaction: *p* = 0.284; light intervention: *p* = 0.708), see Fig. 8, third panel.

#### Visual side effects at the end of each study day

The two light interventions did not differ in asthenopic complaints (active light: 18.23 ± 6.26; placebo light: 17.86 ± 7.23; *t*(42) = 0.598, *p* = 0.553). Both light conditions also had no effect on visual acuity (first light pulse condition: LogMAR 0.05 ± 0.38; second light pulse condition: LogMAR 0.08 ± 0.37; *t*(55) = − 0.457, *p* = 0.650).

## Discussion

The present study examined the effects of intermittent morning bright white light exposure on simple and complex attentional performance. Potential light effects were quantified at the performance, subjective and physiological levels.

Subjects showed improved attentional performance (i.e., they responded faster) under the brighter light pulse and higher corneal illuminance. Consistent with other studies (see, e.g.,^54–57^), lapses in the simple attention task and commission errors in the complex attention task were not affected by light. These results complement the findings of a recently published meta-analysis reporting time-of-day independent light effects on attentional measures^19^.

Importantly, a short-lived faster reaction speed in the simple attention task was observed under the brighter light pulse without habituation (i.e., within each test cycle). However, this effect disappeared in the subsequent period of constant bright light exposure. These results suggest a complex interaction between changing light intensities and simple attentional performance.

In contrast, we observed faster reaction speed in the complex attention task with constant bright light exposure, suggesting that a more demanding attention task involving not only sustained detection but also selection of relevant stimuli might benefit from constant exposure to bright light.

Finally, error rates in both attentional tasks varied across the study day under both light interventions. Specifically, we observed more lapses in the aPVT in test cycle 4 and more commission errors in the GNT in test cycle 5. In contrast to other studies^19^, these results suggest that there is no clear effect of time-of-day on attentional performance error rates.

Neuroimaging studies have shown that bright light exposure acutely alters autonomic nervous system activity by projecting photic information from ipRGCs and suprachiasmatic nuclei to the paraventricular nucleus of the hypothalamus and the locus coeruleus of the brainstem^58,59^. In the present study, increased attentional performance in both attentional tasks was accompanied by increased heart rate variability (as determined by RMSSD), suggesting increased parasympathetic activity during performance of these tasks and confirming the results of reviews showing that higher HRV is associated with higher attentional performance and better self-regulatory abilities^31,32^. However, the present study provides the first evidence, that improved attentional performance and parasympathetic activity may be induced by increased brief exposure to light during the day. Furthermore, research has shown (e.g., from the recent past:^60–62^) that attentive anticipation of a stimulus decelerates heart rate (suggesting a physiological freezing state prior to stimulus onset) and stimulus onset accelerates heart rate to mobilize for a rapid and correct response. Because the heart rate recordings in our study were not strictly synchronized with the processing of the attention tasks (i.e., the time stamps of the ECG data and the attention performance data differed by less than five seconds at the individual level), future studies must confirm that the reported light-induced changes in HRV are phasic responses of the heart and not a general tonic activation of the parasympathetic nervous system. In addition, a review by Lok et al.^16^ also described inconclusive effects of bright light exposure during the day on the autonomic nervous system (i.e., bright light either increased sympathetic and decreased parasympathetic activity, had the opposite effect, or had no effect), which also points to the need for further studies.

Additionally, repetition of the attentional tests affected HRV independently of the light intervention, i.e., HRV was highest in the second test cycle. Moreover, HRV was comparable at the beginning and end of the study days. These results suggest an early physiological adaptation process at the beginning of each study day. They also suggest that repetition of attentional tasks may have increased physiological workload. However, our results do not clearly confirm diurnal physiological effects as reported elsewhere^18^.

Subjective sleepiness-alertness ratings were not altered by bright light exposure in our study, which is in contrast to the results of systematic reviews (see, e.g.,^15,19^). There are at least three possible explanations for this result. Ratings were not recorded at the end of the light pulses, but after performing a longer than usual simple attention task (15 min instead of 10 min). This could have led to mental fatigue and masked the effects of bright light on the subjective sleepiness-alertness scale. Second, because we hypothesized only weak daytime light effects on subjective alertness^15^, our study may have been underpowered. Third, subjects rated their sleepiness-alertness at a rather high m-EDI level (86 lx at eye level) before the onset of the light intervention, which may have also masked the acute subjective alerting effect of bright light exposure. Similar results have been described by Ru et al.^54^.

Subjective ratings of workload at the end of each test cycle decreased slightly on each study day under both study conditions. Moreover, affective states were not altered by bright light exposure. These results give rise to the assumption that improved attentional performance was not moderated by altered perceived workload and affective states.

Consistent with other studies in which the luminance levels in the field of view of study participants were carefully selected, e.g.,^34^, visual comfort and performance in the present study were comparable at both low and high corneal illuminance levels.

### Critical discussion of the study design

To date, a variety of cognitive tasks have been used to quantify non-visual light effects (e.g., sustained attention, selective attention, working memory, and response inhibition^18^). However, attention is fundamental to all higher cognitive functions and human behavior^63^ and is therefore commonly used as an outcome measure in light impact research. Furthermore, performance on tasks with increased cognitive complexity, especially when repeated multiple times in a study protocol, can cause training effects and thus mask potential effects of the light intervention. This is not the case for attentional tasks^64,65^. Therefore, we decided to include one simple and one complex attention task in our study protocol.

Similar to the study by Mager et al.^66^, the presentation of a perceivable light change while performing the simple attention task may have facilitated task processing under both study conditions over a short period of time.

As reviews have shown, see, e.g.,^15,19^, alerting light effects are expectedly small to moderate. Based on a power analysis, we decided to include 56 subjects in our study, which is significantly more than in previous studies.

It’s challenging to define an appropriate but ineffective control condition in light impact research. Usually, weak light stimuli of less than 10 lx are used as contrasting interventions (see^19^). The present study was the first to use an active control intervention, pretending a bright light pulse while subjects performed a simple attentional task, was implemented.

A final methodological aspect seems worth discussing here. To date, studies have used widely varying dimming maneuvers, ranging from switching the light on and off for a few milliseconds^67,68^ to changing light settings over a longer period of time (minutes to hours; see e.g.^13,69,70^). In these studies, the light effects were generally measured under constant light conditions (during or immediately after light exposure). However, the dynamics in alerting light effects, i.e., their immediate effects and persistence, have remained largely unexplored^28^. To address this gap, we decided to implement a study protocol in which the simple attentional task was performed under changing light conditions.

### Study limitations

This study comes with several limitations. First, only acute daytime light effects were investigated. Because light exerts its effects even after exposure ends (see, e.g.,^71,72^), possible carry-over effects on nocturnal sleep and the circadian system (i.e., phase shift and change in amplitude) remain to be elucidated. Second, we have studied light effects on a specific cognitive domain (attention). Therefore, investigating acute daytime light effects on other important work-related executive functions, e.g., task switching and decision making, as well as working memory, remains open for future research. Third, previous research has shown that light in the morning has a stronger alerting effect^18^. Consistent with this finding, we measured light effects from 07:30 AM to 12:30 PM in our study. However, bright light exposure may also be effective in the afternoon (e.g., during the post-lunch dip^73^). Fourth, the spectral composition of light was not changed throughout the study. Moreover, neutral-white light was chosen for task lighting instead of cold-white light (6500 K or higher), as studies have shown that this light color is perceived as more pleasant^74,75^. However, it can be hypothesized that light pulses with high melanopic illuminance (i.e., with more short-wavelength radiation) could have even greater effects on attention. In addition, this measure could further improve the energy consumption of bright light pulses and thus support global sustainability goals. Fifth, visual side effects were measured only after full adaptation to each light intervention once at the end of each study day. However, recordings with higher temporal resolution are needed to quantify acute adverse light effects on visual comfort and acuity^76^. Sixth, light pulses were applied at fixed times of the day and not activated according to individual needs (e.g., when study participants felt inattentive). This limitation of the study could be the starting point for follow-up studies examining the effects of self-selected light pulses during the day. The concept of zonal task lighting would allow to study the effects of personalizable bright light exposure at individual workstations without disturbing colleagues. Seventh, study participants were not asked whether they noticed differences in light settings between the two lighting interventions. Thus, it could still be that the reported light effects were influenced by different perceived light settings. Eighth, the light effects in the complex attention task were recorded only at constant and strongly different corneal illuminance levels and not at the two light pulses. Ninth, study participants were asked to adhere to a specific sleep protocol. Although sleep quality was monitored using a sleep questionnaire, no objective sleep parameters (e.g., actimetry) were recorded. Furthermore, our sample included a wide age range of 20–50 years. With age (usually at an age of 40 years), the human lens becomes increasingly yellow, leading to age-related changes in the input to ipRGCs and color perception. However, in our study, only 4 of the 56 subjects were 40 years of age or older. Because of the small sample, it was not possible to carry out additional age-specific analysis. Finally, this study investigated light effects on a single day. Although the study protocol explored the effects of repeated bright light exposure on single days, humans may adapt to this type of light exposure over several days.

### Conclusions

In conclusion, the present study demonstrates the attention-enhancing effect of repeated brief daytime bright light exposures under laboratory conditions in a healthy sample. The results suggest different underlying mechanisms of action for acute light effects on simple and complex attentional tasks and warrant further basic research to better understand these mechanisms. In particular, we recommend the investigation of light-induced changes in event-related physiological responses (e.g., pupillary dynamics, phasic electrodermal activity, and heart rate responses).

Integrative lighting, as defined by CIE (CIE S 017/E:2020^77^), should include both visual and non-visual aspects of lighting. The results of our study demonstrate that bright light pulses and exposure to increased corneal illuminance during the day can be an effective measure to achieve this goal. In addition, short light pulses consume less energy compared to continuous bright light exposure and therefore could also be a promising energy-efficient countermeasure to decreased daytime attention in the workplace.

## Supplementary Information

Supplementary Information.

## Data Availability

Aggregated data is available from the corresponding author upon request.
