# Supplementary material for: Impact of repeated morning bright white light exposures on attention in a simulated office environment
Source: Sci Rep. 2023 May 30;13:8730. doi: 10.1038/s41598-023-35689-1 (PMC10229615; doi:10.1038/s41598-023-35689-1)
Supplement: Supplementary file 1 — Supplementary Information. [file 41598_2023_35689_MOESM1_ESM.docx]

**Impact of repeated morning bright white light exposures on attention in a simulated office environment**

**Supplementary Materials**

Markus Canazei^1^. Maximilian Dick^1.2^. Wilfried Pohl^2^. Johannes Weninger^2^. Niclas Hubel^1^. Siegmund Staggl^1^. Elisabeth M. Weiss^1^

^1^ Department of Psychology. University of Innsbruck. Innrain 52f. Innsbruck. Austria

^2^ Research and Development Department. Bartenbach GmbH. Rinnerstrasse 14. Aldrans. Austria

Corresponding author:

Department of Psychology. University of Innsbruck

Innrain 52 f. 6020 Innsbruck. Austria

email: [markus.canazei@uibk.ac.at](mailto:markus.canazei@uibk.ac.at)

phone: +43 512 507 56060

fax: +43 512 507 56199

**S1. Spectral data**

| wavelength | standard lighting  (130 lx*) | FLP  (1360 lx*) | SLP  (870 lx*) | SLP  (65 lx*) |
| --- | --- | --- | --- | --- |
| 380 | 0.0000 | 0.0000 | 0.0000 | 0.0000 |
| 385 | 0.0000 | 0.0000 | 0.0000 | 0.0000 |
| 390 | 0.0000 | 0.0000 | 0.0000 | 0.0000 |
| 395 | 0.0000 | 0.0000 | 0.0000 | 0.0000 |
| 400 | 0.0000 | 0.0000 | 0.0000 | 0.0000 |
| 405 | 0.0000 | 0.0001 | 0.0001 | 0.0000 |
| 410 | 0.0000 | 0.0001 | 0.0001 | 0.0000 |
| 415 | 0.0001 | 0.0006 | 0.0004 | 0.0000 |
| 420 | 0.0001 | 0.0014 | 0.0009 | 0.0001 |
| 425 | 0.0003 | 0.0028 | 0.0018 | 0.0001 |
| 430 | 0.0005 | 0.0054 | 0.0035 | 0.0003 |
| 435 | 0.0009 | 0.0096 | 0.0061 | 0.0005 |
| 440 | 0.0015 | 0.0157 | 0.0100 | 0.0008 |
| 445 | 0.0020 | 0.0211 | 0.0135 | 0.0010 |
| 450 | 0.0021 | 0.0217 | 0.0139 | 0.0010 |
| 455 | 0.0018 | 0.0183 | 0.0117 | 0.0009 |
| 460 | 0.0014 | 0.0142 | 0.0091 | 0.0007 |
| 465 | 0.0011 | 0.0114 | 0.0073 | 0.0005 |
| 470 | 0.0009 | 0.0094 | 0.0060 | 0.0004 |
| 475 | 0.0008 | 0.0085 | 0.0055 | 0.0004 |
| 480 | 0.0008 | 0.0087 | 0.0056 | 0.0004 |
| 485 | 0.0009 | 0.0096 | 0.0061 | 0.0005 |
| 490 | 0.0010 | 0.0109 | 0.0069 | 0.0005 |
| 495 | 0.0012 | 0.0121 | 0.0078 | 0.0006 |
| 500 | 0.0013 | 0.0134 | 0.0086 | 0.0006 |
| 505 | 0.0014 | 0.0145 | 0.0093 | 0.0007 |
| 510 | 0.0015 | 0.0155 | 0.0099 | 0.0007 |
| 515 | 0.0016 | 0.0162 | 0.0104 | 0.0008 |
| 520 | 0.0016 | 0.0166 | 0.0106 | 0.0008 |
| 525 | 0.0016 | 0.0172 | 0.0110 | 0.0008 |
| 530 | 0.0017 | 0.0176 | 0.0113 | 0.0008 |
| 535 | 0.0017 | 0.0181 | 0.0116 | 0.0009 |
| 540 | 0.0017 | 0.0182 | 0.0117 | 0.0009 |
| 545 | 0.0018 | 0.0185 | 0.0119 | 0.0009 |
| 550 | 0.0018 | 0.0188 | 0.0120 | 0.0009 |
| 555 | 0.0018 | 0.0191 | 0.0122 | 0.0009 |
| 560 | 0.0018 | 0.0192 | 0.0123 | 0.0009 |
| 565 | 0.0019 | 0.0196 | 0.0125 | 0.0009 |
| 570 | 0.0019 | 0.0198 | 0.0127 | 0.0009 |
| 575 | 0.0019 | 0.0202 | 0.0129 | 0.0010 |
| 580 | 0.0020 | 0.0205 | 0.0131 | 0.0010 |
| 585 | 0.0020 | 0.0208 | 0.0133 | 0.0010 |
| 590 | 0.0020 | 0.0212 | 0.0136 | 0.0010 |
| 595 | 0.0021 | 0.0215 | 0.0137 | 0.0010 |
| 600 | 0.0021 | 0.0217 | 0.0139 | 0.0010 |
| 605 | 0.0021 | 0.0218 | 0.0140 | 0.0010 |
| 610 | 0.0021 | 0.0219 | 0.0140 | 0.0010 |
| 615 | 0.0021 | 0.0218 | 0.0139 | 0.0010 |
| 620 | 0.0021 | 0.0216 | 0.0138 | 0.0010 |
| 625 | 0.0020 | 0.0211 | 0.0135 | 0.0010 |
| 630 | 0.0020 | 0.0207 | 0.0132 | 0.0010 |
| 635 | 0.0019 | 0.0201 | 0.0129 | 0.0010 |
| 640 | 0.0018 | 0.0193 | 0.0123 | 0.0009 |
| 645 | 0.0018 | 0.0186 | 0.0119 | 0.0009 |
| 650 | 0.0017 | 0.0177 | 0.0113 | 0.0008 |
| 655 | 0.0016 | 0.0168 | 0.0107 | 0.0008 |
| 660 | 0.0015 | 0.0159 | 0.0102 | 0.0008 |
| 665 | 0.0014 | 0.0148 | 0.0095 | 0.0007 |
| 670 | 0.0013 | 0.0139 | 0.0089 | 0.0007 |
| 675 | 0.0012 | 0.0129 | 0.0083 | 0.0006 |
| 680 | 0.0011 | 0.0119 | 0.0076 | 0.0006 |
| 685 | 0.0010 | 0.0110 | 0.0070 | 0.0005 |
| 690 | 0.0009 | 0.0099 | 0.0063 | 0.0005 |
| 695 | 0.0009 | 0.0090 | 0.0058 | 0.0004 |
| 700 | 0.0008 | 0.0082 | 0.0052 | 0.0004 |
| 705 | 0.0007 | 0.0073 | 0.0047 | 0.0004 |
| 710 | 0.0006 | 0.0066 | 0.0042 | 0.0003 |
| 715 | 0.0006 | 0.0061 | 0.0039 | 0.0003 |
| 720 | 0.0005 | 0.0053 | 0.0034 | 0.0003 |
| 725 | 0.0005 | 0.0048 | 0.0030 | 0.0002 |
| 730 | 0.0004 | 0.0041 | 0.0026 | 0.0002 |
| 735 | 0.0003 | 0.0036 | 0.0023 | 0.0002 |
| 740 | 0.0003 | 0.0032 | 0.0021 | 0.0002 |
| 745 | 0.0003 | 0.0028 | 0.0018 | 0.0001 |
| 750 | 0.0002 | 0.0026 | 0.0016 | 0.0001 |
| 755 | 0.0002 | 0.0023 | 0.0014 | 0.0001 |
| 760 | 0.0002 | 0.0020 | 0.0013 | 0.0001 |
| 765 | 0.0002 | 0.0019 | 0.0012 | 0.0001 |
| 770 | 0.0002 | 0.0017 | 0.0011 | 0.0001 |
| 775 | 0.0002 | 0.0016 | 0.0010 | 0.0001 |
| 780 | 0.0001 | 0.0013 | 0.0009 | 0.0001 |

Note: * corneal illuminances.

**S2. Full three-factor r-ANOVA with aPVT reaction speed data**

A full three-factor r-ANOVA model was additionally run for the aPVT reaction speed data. The three within-subject factors were: part (‘baseline’, intervention’, and ‘different light’), test cycle (test cycle 1 – 5), and light intervention (‘stronger light pulse followed by bright light exposure’, ‘weaker light pulse followed by standard workplace light exposure’).

The r-ANOVA yielded a significant interaction effect between the two factors ‘part’ and ‘light intervention’, F(2,440) = 3.943, *p =* .024, η²_p_ = .067. The three other interactions tested with this r-ANOVA model (i.e., ‘part’ x ‘test cycle’, ‘light intervention’ x ‘test cycle’, and ‘light intervention’ x ‘test cycle’ x ‘part’) did not reach significance (all *p* > .10).

Pairwise comparisons within each light intervention of the significant interaction between ‘part’ and ‘light intervention’ revealed a significantly higher aPVT reaction speed for the ‘stronger light pulse followed by bright light exposure’ - condition at part ‘baseline’ (3.98 ± 0.12) and part ‘intervention’ (3.94 ± 0.14) compared to part ‘different light’ (3.85 ± 0.12) with *p* < .001 and *p* = .005, respectively. However, the mean reaction speed at part ‘baseline’ and part ‘intervention’ were not different (*p* = .106). Moreover, aPVT reaction speeds for all three parts of the ‘weaker light pulse followed by standard workplace light exposure’-condition were significantly different (all *p* < .005) with the highest reaction speed observed at part ‘baseline’ (3.94 ± 0.14), slower reaction speed at part ‘intervention’ (3.86 ± 0.14) and the slowest reaction speed at part ‘different light’ (3.80 ± 0.14).

**S3. Confounder analysis**

**S3.1. Physiological states during resting periods**

A two-factor r-ANOVA was run for the two cardiovascular parameters RR-intervals and RMSSD derived during the 25-min resting periods for the five resting cycles. We could not observe a significant interaction nor main effect of light intervention for RR-intervals (interaction: *p =* .554; light intervention: *p =* .223). However, the main effect of resting cycle was significant (*F*(4, 220) = 4.240, *p =* .016, η²_p_ = .072). Pairwise comparisons revealed significantly higher RR-intervals in resting cycle 2 (900 ± 28) compared to resting cycle 1 (866 ± 29), *p <* .001, but no other significant differences between the other pairs of resting cycles. Similarly, the interaction effect and the main effect of light intervention was not significant for the HRV-parameter RMSSD (interaction: *p =* .488; light intervention: *p =* .938). Again, the main effect of resting cycle for RMSSD reached significance, *F*(4, 220) = 11.691, *p <* .001, η²_p_ = .173. The RMSSD scores in resting cycle 2 (57.1 ± 5.4) and resting cycle 3 (56.9 ± 5.0) were significantly higher than in resting cycle 1 (49.3 ± 5.0) and resting cycle 5 (52.4 ± 5.1), all *p <* .05.

**S3.2. Comparisons of reaction speed in attentional tasks between the first and second study day**

Two-factor r-ANOVAs for reaction speed of the aPVT, run for each part of the test separately, revealed no significant interactions nor main effects of study day and test cycle (all p > .10). Moreover, a statistical analysis of reaction speed in GNT also showed no significant main effect of study day nor interaction effect between study day and test cycle (both p > .10). However, the main effect of test cycle in GNT reaction speed was significant (F(4, 220) = 3.707, p = .011, η²p = .063), with significantly higher reaction speed in test cycle 5 (3.12 ± 0.10) than test cycle 1 (3.06 ± 0.10) on both study days.

**S4. Gender-specific light effects on the two attention tasks**

For statistical hypothesis testing, three-factor mixed r-ANOVAs with the two within-subjects factor ‘light intervention’ (2 levels: stronger light pulse, weaker light pulse) and the daytime factor ‘test cycle’ (5 levels: test cycle 1 - 5) and the between-subjects factor ‘gender’ (2 levels: female, male) were run separately for each of the three parts (‘baseline’, ‘intervention’, and ‘different light’) of the aPVT reaction speed and GNT reaction speed. Moreover, these tests were also run for the HRV parameter RMSSD. The following two tables summarize the test results.

| Factors | F-statistics | p-value |  |
| --- | --- | --- | --- |
| **aPVT – “baseline” (reaction speed)** | | | |
| Test cycle | 1.001 | .397 |  |
| Test cycle * gender | 0.543 | .663 |  |
| Light intervention | 1.706 | .197 |  |
| Light intervention * gender | 1.976 | .166 |  |
| Test cycle * light intervention | 0.109 | .964 |  |
| Test cycle * light intervention * gender | 0.535 | .675 |  |
| Gender | 0.038 | .846 |  |
| **aPVT – “intervention” (reaction speed)** | | |  |
| Test cycle | .530 | .662 |  |
| Test cycle * gender | .796 | .498 |  |
| Light intervention | 9.563 | .003* |  |
| Light intervention * gender | 2.450 | .123 |  |
| Test cycle * light intervention | 0.387 | .802 |  |
| Test cycle * light intervention * gender | 0.829 | .500 |  |
| Gender | 0.014 | .907 |  |
| **aPVT – “different light” (reaction speed)** | | |  |
| Test cycle | 1.982 | .118 |  |
| Test cycle * gender | 0.697 | .557 |  |
| Light intervention | 3.818 | .056 |  |
| Light intervention * gender | 3.432 | .069 |  |
| Test cycle * light intervention | 0.725 | .574 |  |
| Test cycle * light intervention * gender | 1.178 | .321 |  |
| Gender | 0.002 | .968 |  |
| **GNT (reaction speed)** | | |  |
| Test cycle | 3.738 | .010* |  |
| Test cycle * gender | 0.556 | .695 |  |
| Light intervention | 7.623 | .008* |  |
| Light intervention * gender | 0.072 | .789 |  |
| Test cycle * light intervention | 0.954 | .422 |  |
| Test cycle * light intervention * gender | 0.555 | .660 |  |
| Gender |  |  |  |

Note: * indicates significant result

| Factors | F-statistics | p-value |  |
| --- | --- | --- | --- |
| **aPVT – “baseline” (HRV-parameter RMSSD)** | | | |
| Test cycle | 8.262 | <.001* |  |
| Test cycle * gender | 0.217 | .858 |  |
| Light intervention | 0.848 | .361 |  |
| Light intervention * gender | 1.487 | .228 |  |
| Test cycle * light intervention | 0.164 | .956 |  |
| Test cycle * light intervention * gender | 1.684 | .165 |  |
| Gender | 0.154 | .696 |  |
| **aPVT – “intervention” (HRV-parameter RMSSD)** | | |  |
| Test cycle | 9.699 | <.001* |  |
| Test cycle * gender | 0.269 | .813 |  |
| Light intervention | 5.008 | .029* |  |
| Light intervention * gender | 0.730 | .397 |  |
| Test cycle * light intervention | 0.140 | .948 |  |
| Test cycle * light intervention * gender | 0.288 | .852 |  |
| Gender | 0.387 | .536 |  |
| **aPVT – “different light” (HRV-parameter RMSSD)** | | |  |
| Test cycle | 13.430 | <.001* |  |
| Test cycle * gender | 1.506 | .201 |  |
| Light intervention | 2.081 | .155 |  |
| Light intervention * gender | 0.307 | .582 |  |
| Test cycle * light intervention | 0.124 | .951 |  |
| Test cycle * light intervention * gender | 0.909 | .441 |  |
| Gender | 0.440 | .510 |  |
| **GNT (HRV-parameter RMSSD)** | | |  |
| Test cycle | 7.283 | <.001* |  |
| Test cycle * gender | 0.825 | .490 |  |
| Light intervention | 7.997 | .007 |  |
| Light intervention * gender | 0.310 | .580 |  |
| Test cycle * light intervention | 1.054 | .372 |  |
| Test cycle * light intervention * gender | 0.830 | .483 |  |
| Gender | 0.835 | .365 |  |

Note: * indicates significant result
